# Editorial: ACTH Action in the Adrenal Cortex: From Molecular Biology to Pathophysiology

**DOI:** 10.3389/fendo.2017.00101

**Published:** 2017-06-12

**Authors:** Nicole Gallo-Payet, Antoine Martinez, André Lacroix

**Affiliations:** ^1^Division of Endocrinology, Department of Medicine, Faculté de médecine et des sciences de la santé, Université de Sherbrooke, Sherbrooke, QC, Canada; ^2^Centre de recherche clinique Étienne-Le Bel of the Centre Hospitalier Universitaire de Sherbrooke (CHUS), Sherbrooke, QC, Canada; ^3^GReD – Génétique, Reproduction & Développement, CNRS/INSERM, Université Clermont-Auvergne, Aubière, France; ^4^Division of Endocrinology, Department of Medicine, Centre de Recherche du Centre hospitalier de l’Université de Montréal (CRCHUM), Montreal, QC, Canada

**Keywords:** adrenal cortex, ACTH, cortisol, aldosterone, signaling, MC2R, adrenal tumors, Cushing’s syndrome

**Editorial on the Research Topic**

**ACTH Action in the Adrenal Cortex: From Molecular Biology to Pathophysiology**

With this Research Topic, we want to celebrate 80 years of research on the role of adrenocorticotropic hormone (ACTH) on the adrenal cortex, from the pioneering work by Selye, who introduced the concept of “general adaptation syndrome” in a short Letter to Nature (1). Selye described the general adaptation syndrome, later renamed stress, as a response of the body to demands placed upon it. The system whereby the body copes with stress, the hypothalamo–pituitary–adrenal (HPA) axis, was also first described by Szabo et al. (2). From a historical standpoint, Selye actively avoided using the term “stress” until 1946. It was Walter Cannon who actually developed the term stress in his work relating to the fight-or-flight response in 1914 (2, 3). Because it was clear that most people viewed stress as some unpleasant threat, Selye had to create a new word, “stressor,” in order to distinguish between stimulus and response (3). It is also important to remember that Selye was the first to describe “corticoids” and to propose that glucocorticoids and mineralocorticoids (also named by Selye) regulated not only carbohydrate and electrolyte metabolism, respectively, but also exerted anti- or pro-inflammatory effects (2, 4, 5).

By stimulating adrenal corticosteroids synthesis, the ACTH, first isolated in 1943 (6) and then synthesized in 1960s and 1970s (see the review by Ghaddhab et al.), plays a central role in homeostasis and stress response and thus is a key component of the HPA axis. This Research Topic is a compendium of reviews and original contributions, which summarize classical views, novel findings, and challenges on the various mechanisms involved in ACTH action, from the binding of ACTH to its receptor to steroid secretion. The Research Topic also reviews the pathological consequences of the disruption of specific components of these pathways.

The adult mammalian adrenal cortex is composed of three concentric layers, each having specific functional and morphological properties. ACTH is the main stimulus of the zona fasciculata and zona reticularis, stimulating glucocorticoids secretion, while angiotensin II and potassium are the main stimuli of aldosterone secretion by the zona glomerulosa. Glucocorticoids (cortisol, corticosterone, cortisone) are implicated in a broad range of metabolic functions, including anti-inflammatory responses, stress response, and behavior. However, chronically elevated glucocorticoid levels alter body protein synthesis, fat distribution, increase visceral adiposity, and are responsible for metabolic abnormalities, such as hypertension, type 2 diabetes, or sleep disturbance. On the other hand, aldosterone stimulates sodium reabsorption, hence maintaining blood volume and pressure in sodium-depleted conditions. Excessive aldosterone secretion not only leads to hypertension and electrolyte imbalance but is also associated with much increased risks of cardiometabolic complications as compared to similar levels of essential hypertension. As reviewed by Funder and El Ghorayeb et al., there is recent evidence that aldosterone secretion is highly sensitive to very low doses of ACTH and that alteration in melanocortin 2 receptor (MC2R) expression is observed in adrenal tissues of patients with primary aldosteronism. Together, these results indicate that ACTH may be an important mediator of inappropriate hypersecretion of aldosterone in this very prevalent disease (approximately 10% of humans with hypertension).

From the recent findings on a number of proteins involved in functional activity of the cortex, Vinson discusses the significance of the standard model of morphological zonation vs. functional zonation. Lerario et al. detail how adrenocortical cells are homeostatically derived from a pool of progenitors localized in the subcapsular region of the zona glomerulosa. These cells undergo a continuous process of centripetal migration. In their review, these authors summarize established and emerging concepts that regulate the biology of the progenitor cell niche. An emphasis is placed on the interactions between extracellular matrix (ECM) components and their cell surface receptors, and with the ECM-embedded signaling molecules, including ligands of the sonic hedgehog and Wnt signaling pathways. Lotfi and de Mendonca review the cascade of events involved in the regulation of proliferation and growth by ACTH and other POMC-derived peptides. Key findings regarding signaling pathways and modulation of genes and proteins required for the regulation of murine adrenal growth are summarized. It is also well known that there are sex differences in adrenal cortex structure and function. In the rat, they are manifested as larger adrenal cortex and higher corticosterone secretion in females compared with males. Jopek et al. have investigated the transcriptome profile of rat adrenal after gonadectomy and testosterone or estradiol replacement. As developed in his article, results were unexpected and the physiological relevance of these findings are discussed.

Dumbell et al. review the importance of circadian clocks and their interactions with the immune system. The review highlights the importance of timing in treatment of many chronical disorders with a chronic inflammatory background. In particular, as documented by Engeland et al., the effect of stress differs depending on the time of day when acute stress is administered. How stress affects circadian rhythms of glucocorticoids secretion and their impact on health is reviewed by Nicolaides et al. Accumulating evidence suggests that dysfunction of the former may result in dysregulation of the latter, and *vice versa*. The authors review the functional components of the two systems and discuss their multilevel interactions during excessive or prolonged activity of the HPA axis. In particular, as discussed by Moeller et al., disruption of circadian timing, such as after inter-time zone travel, shift work, and mistimed eating, can have consequences for cardiovascular, metabolic, and mental health and, crucially, immune function.

Adrenocorticotropic hormone exerts its role through binding to the G protein-coupled receptor, MC2R, which activates the adenylyl cyclase cascade leading to cAMP production and subsequent activation of cAMP-dependent protein kinase A (PKA). PKA is the main kinase responsible for the phosphorylation of specific transcription factors, which in turn regulate free cholesterol availability and activate the expression of steroidogenic enzymes. Six groups detail the current knowledge surrounding the mechanisms of ACTH binding to its receptor. The MC2R is unusual in that it is dependent on a small accessory protein, melanocortin receptor accessory protein (MRAP), which is essential for both trafficking of MC2R to the plasma membrane and for ACTH binding and activation of MC2R (reviewed by Clark et al., Ghaddhab et al., and Fridmanis et al.). MRAP is a single chain protein with one membrane-spanning domain. Thanks to an elegant tool, Maben et al. present new evidence on the dual topology of MRAP. The publication supports previous findings of an antiparallel homodimer structure for MRAP and indicates that partners of the MRAP dimer maintain a fixed orientation during trafficking and integration in the plasma membrane. In the human genome, there are two *MRAP* paralog *MRAP* genes. Some features of this gene family are unique. Dores addresses new hypotheses on the evolution of MCRs and MRAPs, highlighting important considerations on the origin of these key proteins in stress axis in vertebrates. One hypothesis is that differences in affinity between MCR–MRAP interactions may affect the trafficking of certain receptors to the cell membrane or allow activation of the receptor by its ligand. In their review, Maben et al. and Fridmanis et al. detailed how specific mutations in the region of the N-terminal tail of MRAP1 and MRAP2 are essential for promoting only the trafficking of receptors to the plasma membrane (MRAP2) or essential for ACTHR/MC2R ligand recognition and functionally (MRAP1).

Even if the structure of ACTH has been known for many years, the exact mechanism of activation of MC2R by ACTH is still unknown. ACTH is the only known naturally occurring ligand for this receptor. The lack of redundancy and high degree of ligand specificity suggests that antagonism for this receptor could provide a useful therapeutic tool for some difficult-to-treat diseases such as congenital adrenal hyperplasia and primary bilateral macronodular adrenal hyperplasia (BMAH), as well as in Cushing’s disease or stress-related pathologies. In their reviews, Clark et al. and Ghaddhab et al. discuss the scientific and clinical rationale for the development of an ACTH receptor antagonist. However, as reviewed by the two groups, the design of a specific peptide acting as an MC2R antagonist remains a challenge due to the difficulty of designing a tridimensional peptide specific for only one of five MCR receptors. Indeed, the first 13 residues of ACTH are active on all the other melanocortin receptors and thus it seems that this tetrabasic region acts as a key to unlock the MC2R–MRAP complex. In addition, all melanocortin receptors share the same amino acid sequences H6F7R8W9 (the “message sequence”), which is important for melanocortin receptor stimulation and which bind to all MCR receptors, while a.a. 15–19 (KKRRP sequence) (the “address sequence”), is essential for the binding of ACTH to MC2R. If they are not selective, these peptides could be source of severe secondary effects due to numerous roles of five MCRs. Moreover, a small, non-peptide molecule that can be delivered orally would be more desirable. Interestingly, in neonates, Nensey et al. detailed that stress-induced increases in corticosterone demonstrate a unique shift from ACTH independence at postnatal day 2 (PD2) to ACTH dependence at day 8 (PD8). If the corticosterone response to stress in PD2 pups can be blocked by antagonizing the MC2R, it would suggest that a bioactive form of ACTH (not measured by immunoassay) is working through the MC2R in PD2 pups. Nensey et al. describe the effect of two novel MC2R antagonists, GPS1573 and GPS1574, on the corticosterone response to ACTH in these two neonatal stages of development.

Several reviews and original contributions develop recent advances on the mechanisms by which ACTH stimulates steroidogenesis. The acute response of ACTH is initiated by the mobilization of cholesterol from lipid stores (Shen et al.) and its delivery to the inner mitochondrial membrane (Lee et al. and Midzak and Papadopoulos), a process that is mediated by the steroidogenic acute regulatory protein (StAR) (Clark), while the chronic response results in the increased coordinated transcription of genes encoding steroidogenic enzymes (Ruggiero and Lalli). One of the initial events of ACTH action is the regulation of expression and function of scavenger receptor class B type I (SR-B1). Shen et al. review how both acute and chronic ACTH treatments can modulate transcription, posttranscriptional stability, phosphorylation, and dimerization status of SR-B1, as well as the interaction of SR-B1 with other protein partners, all of these being important for SR-B1 to mediate lipoprotein-derived cholesterol esters uptake and the supply of cholesterol to mitochondria. Once cAMP is stimulated, activation of the cAMP-dependent PKA results in an acute increase in expression and activity of StAR. As reviewed by Clark, StAR plays an essential role in steroidogenesis in coordinating ACTH-dependent movement of cholesterol across mitochondrial membranes. The model for StAR translation and phosphorylation at the outer mitochondrial membrane, the site for StAR function, is presented. A model for the spatiotemporal regulation of StAR by cAMP is presented by Lee et al. The C-terminal cholesterol binding domain of StAR initiates mitochondrial inter-membrane contacts to rapidly direct cholesterol to the cholesterol side chain cleavage enzyme (Cyp11a1) in the inner mitochondrial membrane. The conserved StAR N-terminal regulatory domain (NTRD) controls the transit time that determines extra-mitochondrial StAR effects on cholesterol homeostasis. On the other hand, Midzak and Papadopoulos highlight the functional importance of the assembly of large multimeric protein complexes in mitochondrial cholesterol transport, steroidogenesis, and mitochondria–endoplasmic reticulum contact. The complex includes more than 10 proteins crossing the outer and inner mitochondrial membranes. The importance of StAR in steroidogenesis is illustrated by two cases of lipoid congenital adrenal hyperplasia (LCAH) due to homozygous *StAR/STARD1* mutations. Khoury et al. review the impact of these mutations on steroidogenesis and fertility in LCAH patients. Their publication examines the correlation between the molecular structure of STARD1 with its function in all steroidogenic tissues. Ruggiero and Lalli review the impact of ACTH signaling on transcriptional regulation of steroidogenic genes. They provide a general view of the transcriptional control exerted by the ACTH/cAMP system on the expression of genes encoding steroidogenic enzymes in the adrenal cortex. A special emphasis is put on the transcription factors required to mediate ACTH-dependent transcription of steroidogenic genes. On the other hand, the regulation of mRNA stability has emerged as a critical control step in dynamic gene expression. This process occurs in response to modifications of the cellular environment, including hormonal variations. In the adrenal cortex, Desroches-Castan et al. explain how ACTH or cAMP elicit changes in the expression, phosphorylation, and localization status of major mRNA-destabilizing protein.

As reviewed by Paz et al., steroidogenesis depends not only on PKA-mediated Ser/Thr phosphorylation but also on the activity of protein tyrosine phosphatases, which have been implicated in StAR expression and steroidogenesis. Furthermore, arachidonic acid and its metabolites play a key role in the hormonal control of steroidogenesis by regulating both the expression and function of StAR. The role of exchange protein activated by cAMP (EPAC) and the actin cytoskeleton in cAMP-mediated actions of ACTH is updated by Lewis et al. In adrenocortical cells in culture, cAMP induces characteristic changes in cell shape and a concomitant reorganization of actin microfilaments. This cytoskeletal reorganization allows the correct positioning of lipid droplets, endoplasmic reticulum, and mitochondria where cholesterol and its metabolites are transported and metabolized. However, the effects of EPAC on F-actin remodeling are not correlated to steroidogenesis and may, therefore, contribute to other aspects of adrenal physiology. Their findings indicate that EPAC2B has a role in cell motility, suggesting rather an implication in adrenal pathophysiology, such as adrenal cancer cell invasion. Finally, in addition to the cAMP–PKA pathway, multiple interactions exist between various signaling cascades. Spat et al. summarize the positive cross talk interactions between various signaling pathways, including calcium and cAMP, on gene expression, mitochondrial function, and thus on steroid secretion. In particular, the contribution of the various calcium channels expressed in adrenocortical cells to steroid production is reviewed. Rossier details the involvement of T-type and L-type calcium channels in the regulation of intracellular calcium concentration and aldosterone secretion in zona glomerulosa cells. In parallel with steroidogenesis, Pastel et al. review the importance of aldo-keto reductases 1B (AKR1B) in adrenal cortex physiology. Adrenal activities generate large amounts of harmful aldehydes from lipid peroxidation and steroidogenesis, which can both be reduced by AKR1B proteins. In addition, chronic effects of ACTH result in a coordinated regulation of genes encoding the steroidogenic enzymes and some AKR1B isoforms. The review describes the molecular mechanisms accounting for the adrenal-specific expression of some AKR1B genes. Using data from recent mouse genetic models, the authors establish a connection between the enzymatic properties of these AKR1B proteins and the regulation of adrenal functions.

The functional activity of the adrenal cortex is the result of an integration of not only cell-specific regulatory processes but also of the paracrine interactions between adjacent cells, as well as paracrine factors, such as ACTH, locally produced in the medulla. How ACTH interacts with the adrenal microenvironment to modulate corticosteroid secretion was reviewed by Bell and Murray who described the fundamental concepts and implication of gap junctions in steroidogenesis, cell proliferation and cancer. For instance, cells of the zona glomerulosa, which have fewer gap junctions than the two other cortical zones, may be less dependent on cell–cell communication for normal function than the cells of the zona fasciculata or the zona reticularis. In their review, Kanczkowski et al. discuss the importance of the adrenal gland microenvironment in the regulation of stress-induced hormone secretion. Among these, adrenocortical–chromaffin cell interactions, the immune adrenal cross talk and the adrenal vascular system play major roles. Lefebvre et al. highlight the importance of ACTH in the interactive and paracrine regulation of adrenal steroid secretion in physiological and pathophysiological conditions. Indeed, ACTH has been found to be abnormally synthesized in bilateral macronodular adrenal hyperplasia (BMAH) responsible for hypercortisolism. In these nodules, ACTH is detected in a subpopulation of adrenocortical cells that express gonadal markers.

Finally, the Research Topic reviews the pathological consequences of a defect in ACTH signaling. Chronic ACTH excess leads to chronic cortisol excess. In the review by Bertagna, all the pathological situations involving an increase in ACTH are reviewed. Some of these include Cushing’s syndrome, excess adrenal androgens, chronic adrenal mineralocorticoid excess, and low aldosterone levels. Indeed, in human, chronic ACTH treatment may induce a sustained increase in cortisol and 11 deoxycorticosterone (two products for the zona fasciculata), in parallel with the low aldosterone levels, eventually creating a state of chronic mineralocorticoid excess. Prolonged *in vivo* stimulation with ACTH, through the activation of a local and complex network of autocrine growth factors, including non-ACTH POMC-peptides, may also affect cell proliferation and hyperplasia, thus participating in the amplified response of the chronically stimulated gland, and the weight of each gland can be greatly increased.

As reviewed by Funder, aldosterone may be acutely stimulated by ACTH that could thus play a role in driving inappropriate aldosterone secretion, as detailed in some examples. El Ghorayeb et al. review the role of ACTH and other hormones in the regulation of aldosterone production in primary aldosteronism, focusing on the aberrant expression of MC2R compared to other GPCRs and their ligands. Alterations in the cAMP-dependent signaling pathway have been implicated in the majority of benign adrenocortical tumors (ACTs) causing Cushing’s syndrome. Villares Fragoso et al. revisit how *GNAS (gsp*) mutations were associated with adrenal cortical tumors and hyperplasia. Although somatic activating mutations in *GNAS* are a rare molecular event in bilateral macronodular hyperplasia, these mutations have recently been identified in a approximately 10% of cortisol-secreting unilateral adenomas. Phosphodiesterases (PDEs) are enzymes that regulate cyclic nucleotide levels, including cAMP. As reviewed by Hannah-Shmouni et al., inactivating mutations and other functional variants in PDE11A and PDE8B, encoding cAMP-binding PDEs, predispose to ACTs. Seidel and Scholl review the molecular differences between aldosterone- and cortisol-secreting adrenal cortical adenomas (APAs and CPAs) leading to primary aldosteronism and Cushing’s syndrome, respectively. In both cases, acute stimulation leads to increased hormone production, and chronic stimulation causes hyperplasia of the respective zones. The authors review some of the pathologies (APAs and CPAs) caused by channel mutations (referred to as channelopathies), for example, mutations in the K^+^ channel gene, *KCNJ5*, or mutations in the Ca^2+^ channel gene, *CACNA1D*, which directly lead to increased calcium influx. Some CPAs carry a recurrent gain-of-function mutation in the *PRKACA* gene, which causes constitutive PKA activity. The contribution by Leccia et al. reviews the mouse models recapitulating human ACTs, highlighting their limitations in replicating human diseases. Development of mouse models is a crucial step to firmly establish the functional significance of candidate genes, to dissect mechanisms leading to tumors and endocrine disorders and to provide *in vivo* tools for therapeutic screens. In the article, the authors provide an overview on the existing mouse models of ACTs by focusing on the role of PKA and Wnt/β-catenin pathways in this context. They present the advantages and limitations of models that have been developed and point out to the necessary improvements for the development of the next generation of mouse models of adrenal diseases.

Two examples of pathological stressful situations due to ACTH and glucocorticoids excess are given in the reviews by Annane and Moeller et al. Annane revisits the role of ACTH and glucocorticoids in septic shock and sepsis. Sepsis is a common disorder associated with high morbidity and mortality. It is now defined as an abnormal host response to infection, resulting in life-threatening dysfunction of organs. Numerous experimental and clinical data have established the paramount importance of an appropriate activation of the HPA axis to respond to severe infection. Clinical trials in sepsis found variability in beneficial effects of corticosteroids on survival, although most trials demonstrated faster resolution in shock and organ dysfunction, suggesting that physicians should consider corticosteroids mainly in septic shock who do not respond rapidly to fluid therapy and vasopressors. Moeller et al. examine how glucocorticoids regulate the food choice behavior in humans with evidence from Cushing’s syndrome. The review is an attempt to elucidate the contribution of glucocorticoid to food-seeking behavior and reward processing, aiming to understanding the mechanistic of weight fluctuations associated with oral glucocorticoid therapy and/or chronic stress.

Even if many questions have been answered since the seminal pioneering work by Selye, including the cloning of the ACTH receptor, MC2R, in 1992 (7) and the discovery of MRAP, about 10 years ago, in 2005 (8), many of the aspects addressed in this Research Topic still represent a stimulus for future studies. These topics reinforced the evidence that the adrenal cortex, through the steroid hormones it secretes, occupies a central position in many metabolic functions where if its homeostasis disrupted have profound deleterious effects. An in-depth investigation of the mechanisms underlying these pathways will be invaluable in developing new therapeutic tools and strategies.

## Author Contributions

As guest editor, I and my co-editors, AL and AM, have read all the contributions, selected external reviewers, reviewed the forum discussion, and finalized decisions regarding final acceptability for publications.

## Conflict of Interest Statement

The authors declare that the research was conducted in the absence of any commercial or financial relationships that could be construed as a potential conflict of interest.
